# CYP2C8 and CYP2C9 mRNA expression profile in the human fetus

**DOI:** 10.3389/fgene.2014.00058

**Published:** 2014-03-25

**Authors:** Maria Johansson, Emmanuel Strahm, Anders Rane, Lena Ekström

**Affiliations:** Division of Clinical Pharmacology, Department of Laboratory Medicine, Karolinska InstitutetStockholm, Sweden

**Keywords:** CYP2C8, CYP2C9, fetus, drug metabolism, ibuprofen

## Abstract

CYP2C8 and CYP2C9 are involved in the inactivation of several non-steroidal anti-inflammatory drugs, including ibuprofen. CYP2C9 is the major form in human liver whereas CYP2C8 has been proposed to be the main CYP2C enzyme in fetal liver. The protein expression of CYP2C9 in the first trimester is low, only about 1% of the adult values, whereas the mRNA levels of CYP2C8/9 have not been determined at the fetal stage. In this study the mRNA expression levels of CYP2C8 and CYP2C9 were determined in 20 adult and 60 fetal liver tissue specimens. The expression profiles in fetal kidneys (*n* = 43), adrenals (*n* = 46), and lungs (*n* = 37) were also determined. Moreover the activity against ibuprofen hydroxylation was determined in fetus and adult liver microsomes. Adult liver samples expressed 140 and 400 times higher levels of CYP2C8 and CYP2C9 mRNA, respectively, as compared to fetal liver samples. Consistent with this, the hydroxylation of ibuprofen was 40 times higher in the adult liver microsomes. Hepatic CYP2C8 mRNA was three times more abundant than CYP2C9 mRNA in the fetus. Moreover, CYP2C8 were consistently expressed in all fetal tissues investigated, whereas CYP2C9 gene expression was confined to the liver in fetuses. Our results indicate that CYP2C8 plays a more important physiological role than CYP2C9 in the first trimester.

## INTRODUCTION

Non-steroidal anti-inflammatory drugs (NSAIDs) are indicated for the treatment of common conditions such as pain, headache, cold, and flu. Many of the NSAIDs are available without a prescription and consequently they are one of the most commonly used drugs during pregnancy (Werler et al., 2005; Ofori et al., 2006). Thus, ibuprofen has been reported to be used among pregnant women in about 15% (Glover et al., 2003; Werler et al., 2005). NSAIDs cross the placenta but what and if any effects are exerted in fetal tissue is not known. It has been proposed that NSAIDs, early in pregnancy, increase the risk of having a child with congenital anomalies and the risk of spontaneous abortion (Ofori et al., 2006; Hernandez et al., 2012). The effects on the ductal artery warrant a caveat against its use close to delivery.

Several of the NSAIDs, including ibuprofen are metabolized by CYP2C8 and CYP2C9. In fact, about 20% of all clinically important drugs are metabolized by members of the CYP2C subfamily (Goldstein, 2001). Additionally both CYP2C8 and CYP2C9 are involved in the metabolism of endogenous substrates such as epoxyeicosatrienoic acids (EETs) and retinoic acids (RAs; Marill et al., 2000; Zeldin, 2001). Despite the structure homology, CYP2C8 and CYP2C9 do not share many substrates, a few are described in the literature including ibuprofen (Totah and Rettie, 2005). CYP2C9 is the major form in human liver (Goldstein and de Morais, 1994) whereas CYP2C8 has been proposed to be the main CYP2C enzyme in fetal liver (Hakkola et al., 1994). The protein expression of CYP2C9 in the first trimester is low, only about 1% of the adult values (Hines, 2007). Hines also described an increase in CYP2C9 expression during the third trimester to 10% of the adult values (Hines, 2007).

Few studies have determined the mRNA expression of CYP2C8 and CYP2C9 in fetus. The purpose of the current study was to determine and compare intra- as well as inter-individual degrees of mRNA expression of CYP2C8 and CYP2C9 in specimens of human fetal (*n* = 60) and adult (*n* = 20) livers, as well as the ibuprofen hydroxylation activity in pooled liver microsomal samples. The mRNA expression profiles of the CYP2C8/9 were also studied in extra-hepatic tissues of the fetuses, in lungs, adrenals, and kidneys.

## MATERIALS AND METHODS

### BIOLOGICAL MATERIAL

Human adult liver specimens (*n* = 20) were collected between 1997 and 2001 as previously described (von Bahr et al., 1980). The study cohort used here included 20 adult livers of male (*n* = 9), female (*n* = 10), or unknown sex (*n* = 1) from Caucasian (*n* = 17) and Asian (*n* = 3) patients between 30 and 75 years of age.

Human fetal liver (*n* = 60), adrenal (*n* = 46), kidney (*n* = 43), and lung (*n* = 37) specimens from 60 fetuses with unknown ethnicity were obtained at legal abortions performed for socio-medical reasons at the Karolinska University Hospital between year 2000–2003. The fetal tissues were excised and immediately frozen in liquid nitrogen and stored at -70°C within 2 h. The study was approved by the Ethics Review Board in Stockholm and by the National Board of Health and Welfare.

The gestational age of the fetuses were determined by crown-rump length and ranged from 5 to 12 weeks (median age 10.2 weeks). The maternal age ranged from 18 to 43 years (median age 29). The sex of the fetuses was not determined at collection since this is not normally documented at abortions and may even be difficult to determine at this early gestational age. None of the women reported any chronic or acute diseases, regular drug use, or drug abuse. Smoking was reported in 22 women (37%), non smoking in 21 (35%), whereas for 17 there were no reports.

### RNA EXTRACTION AND cDNA SYNTHESIS

Total RNA from 5 to30 mg of fetal tissue samples and 200 mg adult liver tissue was prepared using AllPrep DNA/RNA Mini Kit and RNeasy kit (Qiagen, Hilden, Germany), respectively, according to the manufacturer’s protocols. RNA (0.5 μg) was reverse transcribed into cDNA with random hexamers using SuperScript^®^III First Strand Synthesis Super Mix (Invitrogen) according to the manufacturer’s instruction, and diluted 10 times.

### QUANTITATIVE REAL-TIME PCR

Quantitative Real-Time PCR was performed using the 7500 Fast Real-Time PCR System (Applied Biosystems, Foster City, CA, USA). Each PCR reaction mixture had a final volume of 15 μl containing (2×) TaqMan PCR Master Mix (Applied Biosystems), (20×) premade primer and probe mix for CYP2C8 or CYP2C9 (Hs00946140_g1 and Hs01682803_mH, Applied Biosystems), and 1 μl cDNA template. 18S (PN 4310893E, Applied Biosystems) was chosen as an endogenous housekeeping control gene.

The real-time PCR profile for the assay was: 50°C for 2 min, followed by activation at 95°C for 10 min and 40 cycles of denaturation at 95°C for 15 s and annealing/elongation at 60°C for 1 min. Each sample was analyzed in duplicates and no-template controls were included in each experiment. The cycle threshold (Ct) values provided from the real-time PCR were analyzed using the 2^-^^Δ^^Δ^^Ct^ method (Livak and Schmittgen, 2001). One sample was used as calibrator and the mRNA expression is expressed relative the endogenous housekeeping control gene.

### CYP2C9 ENZYME ACTIVITY IN FETAL AND ADULT LIVER MICROSOMES

A pool each of fetal liver microsomes (*n* = 4, 10–12 weeks of age) and adult liver microsomes (*n* = 4, 55–68 years of age) were prepared as described previously (Hakkola et al., 1994). Protein concentration in liver microsomes was measured according to Lowry et al. (1951). The incubation method was based on Chang et al. (2008). Briefly, *S*-ibuprofen was incubated with human liver microsomes (1 mg/mL protein equivalent) in potassium phosphate buffer 0.1 M pH 7.4 and MgCl_2_ 2.5 mM for a total volume of 200 μL. After a pre-incubation at 37°C for 3 min. NADPH (2.4 mM) was added and the mixture was incubated at 37°C for 20 min. The reaction was stopped with 100 μL glacial acetic acid and the *S*-ibuprofen and 2-OH-ibuprofen were extracted by liquid–liquid extraction using 2 mL x 4 mL ethyl acetate/heptanes (50:50, v/v). The organic phase was isolated, evaporated to dryness and reconstituted in 50 μL acetonitrile:H_2_O (20:80, v/v) prior to analysis by liquid chromatography coupled to mass spectrometry detection (LC–MS).

The LC–MS analysis was performed on an Agilent 1100 system from Agilent Technologies (Santa Clara, CA, USA) equipped with a Kinetex C18 column (50 mm x 2.1 mm, 2.6 μm) from Phenomenex (Torrance, CA, USA). A gradient method was used with formic acid 25 mM and acetonitrile, the flow was 0.5 mL/min, the injection volume 1–2 μL and the run time was 5 min. In order to facilitate the determination, three ions were used for the detection of ibuprofen (*m/z* 207 and 224 in the positive mode and *m/z* 205 in the negative mode) and summed up for calculation. Ratios between ions were compared to control the peak purity. The same strategy was applied for 2-OH-ibuprofen with *m/z* 205 and 240 in the positive mode and *m/z* 221 in the negative mode.

Incubations were prepared in triplicates and the activity of one of the adult sample was used as a calibrator.

### DATA ANALYSIS

Statistical analysis was performed using GraphPad Prism software version 4.0 (San Diego, CA, USA). The mRNA expressions in adult samples compared to fetal samples were compared using the Mann–Whitney *U*-test. Kruskal–Wallis followed by Dunn’s test was used for the tissue comparison of mRNA expression. The correlation analyses were performed using the Spearman rank method. Values of *p* < 0.05 were considered to be significant. Values are presented as mean ± SD.

## RESULTS

### CYP2C8 AND CYP2C9 mRNA EXPRESSION IN ADULT AND FETAL LIVER

**FIGURE 1:**
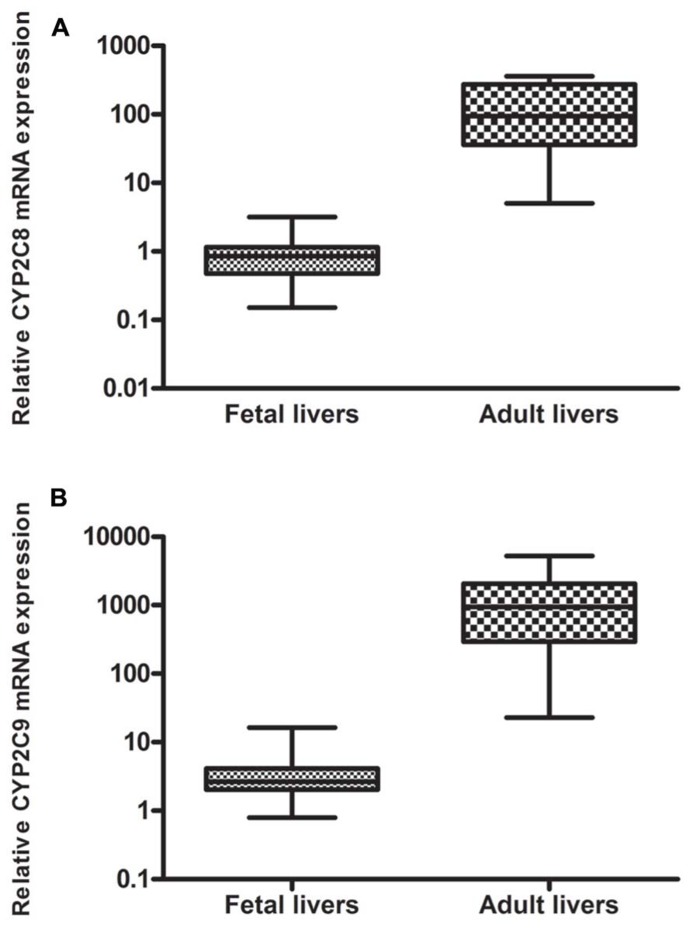
**Relative mRNA expression of (A) CYP2C8 and **(B)** CYP2C9 in liver from human fetuses (*n* = 60) and adults (*n* = 20): Significantly higher levels (143 times) of **(A)** CYP2C8 were observed in the adult liver samples, *p* < 0.0001 and significantly higher levels (396 times) of **(B)** CYP2C9 were observed in the adult liver sample, *p* < 0.0001**.

The mRNA expression of CYP2C8 and CYP2C9 was studied in 20 human adult and 60 fetal liver specimens using quantitative real-time PCR. CYP2C8 mRNA expression was detectable in 96% (*n* = 58) of the fetal liver samples and in 90% (*n* = 18) of the adult liver samples. Significantly higher levels of CYP2C8 were observed in the adult liver samples (mean 136.1 ± 122.0) compared to the fetal liver samples (mean 0.95 ± 0.64), *p* < 0.0001. The mRNA expression of CYP2C8 in fetuses was 0.70% of the adult values (143 times higher in adult compared to fetus; **Figure 1A**).

The mRNA expression of CYP2C9 was detected in 92% (*n* = 55) of the fetal liver samples and in 90% (*n* = 18) of the adult liver samples. Significantly higher expression of CYP2C9 was detected in the adult liver samples (mean 1385 ± 1499) compared to the fetal liver samples (mean 3.54 ± 3.11), *p* < 0.0001. The mRNA expression of CYP2C9 in fetuses was 0.25% of the adult values (396 times higher in adult compared to fetus; **Figure 1B**).

**FIGURE 2:**
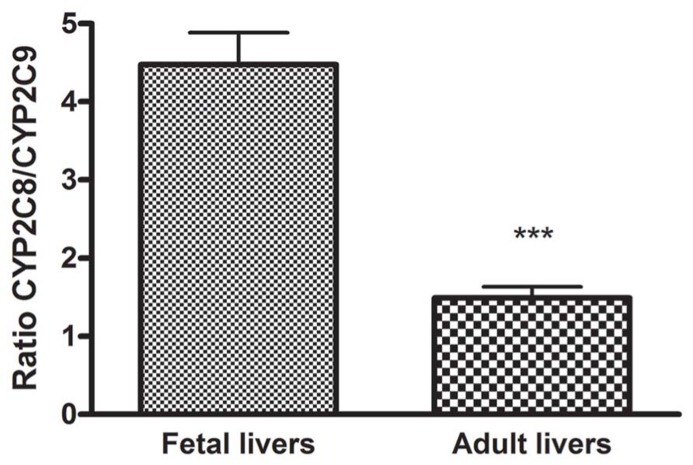
**The ratio of the mRNA expression of CYP2C8 compared to CYP2C9 in fetal and adult liver.** The ratio between CYP2C8 and CYP2C9 was calculated to 4.5 in fetus and 1.5 in adult demonstrating three times higher expression of CYP2C8 compared to CYP2C9 in fetus than in adult. ***p < 0.001.

The ratio between CYP2C8 and CYP2C9 was 4.5 in fetal livers and 1.5 in adult livers demonstrating three times higher expression of CYP2C8 compared to CYP2C9 in fetuses than in adults (**Figure 2**).

A significant difference between the mRNA expression of CYP2C8 (mean 0.95 ± 0.64) compared to CYP2C9 (mean 0.29 ± 0.25) was observed in liver from fetus (*p* < 0.0001) but no difference between these mRNA expressions in adult liver was found (CYP2C8 mean 136 ± 122, CYP2C9 mean 112 ± 121).

**FIGURE 3:**
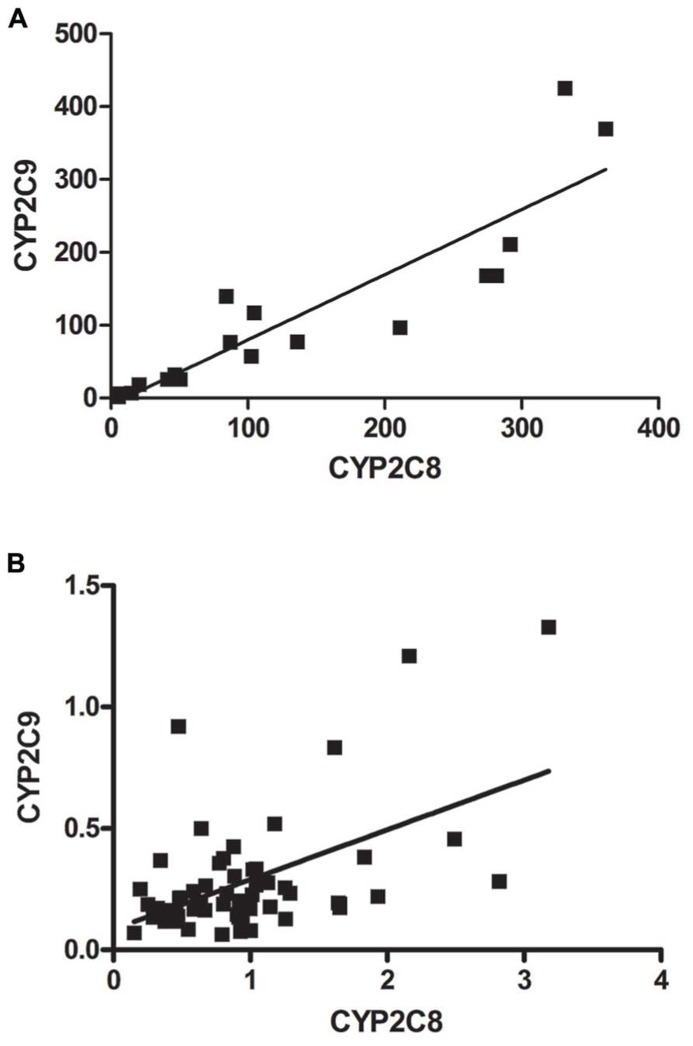
**Relative mRNA expression of CYP2C8 correlated with the relative mRNA expression of CYP2C9 in (A) adult liver and (B) fetal liver**. The correlation study shows a significant association between the mRNA expression of CYP2C9 and CYP2C8 in **(A)** adult liver (*r* = 0.8962, *p* < 0.0001) and in **(B)** fetal liver (*r* = 0.3844, *p* = 0.0038).

The mRNA expression of CYP2C8 and CYP2C9 were significantly correlated in liver in both adults (*r* = 0.8962, *p* < 0.0001) and fetuses (*r* = 0.3844, *p* = 0.0038; **Figures 3A,B**).

The relationship between smoking/maternal age/gestational age and the mRNA expression of CYP2C8/CYP2C9 was also investigated. The only significant correlation found was between the fetal hepatic CYP2C9 mRNA expression and the gestational age of the fetus (*r* = 0.3060, *p* = 0.0364).

### CYP2C9 ENZYME ACTIVITY IN LIVER MICROSOMES FROM ADULT AND FETAL LIVERS

**FIGURE 4:**
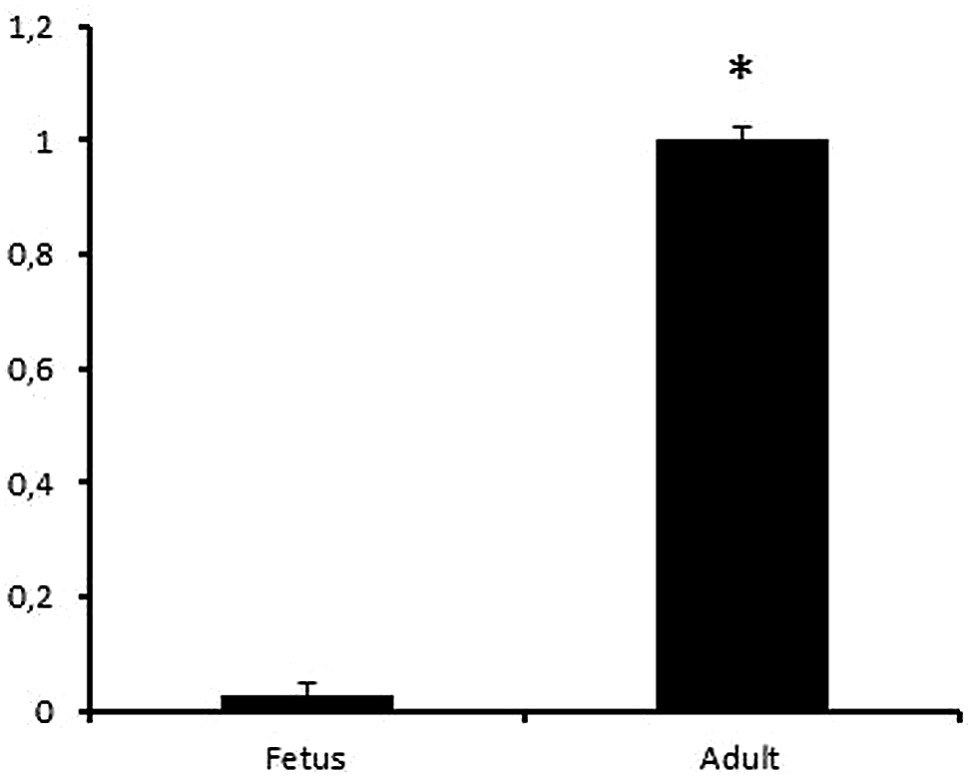
**Relative activity of 2-OH-ibuprofen in fetal and adult liver samples.** Human liver microsomes obtained from pooled adult (*n* = 3) and fetal (*n* = 3) livers were incubated with ibuprofen. The analyses were performed in triplicates. The formation of 2-OH-ibuprofen was significantly higher in adult compared to fetal samples, (*p* = 0.03). *p < 0.05.

The formation of 2-OH-ibuprofen was analyzed using LC–MS after incubation with *S*-ibuprofen for 20 min. The hydroxylation activity was 40 times higher in the adult microsome pool sample (*n* = 3) as compared to the fetal liver microsome pool sample (*n* = 3), *p* = 0.03, (**Figure 4**).

### FETAL EXTRA-HEPATIC CYP2C8 AND CYP2C9 mRNA EXPRESSION

The mRNA expression of CYP2C8 and CYP2C9 were analyzed in liver (*n* = 60), kidney (*n* = 43), adrenal (*n* = 46), and lung (*n* = 37) tissues from fetuses.

CYP2C8 mRNA expression was detected in all of the extra-hepatic tissue samples from fetuses. CYP2C9 mRNA expression was only detected in one of the kidney samples, two of the adrenal samples, and in none of the lung samples.

**FIGURE 5:**
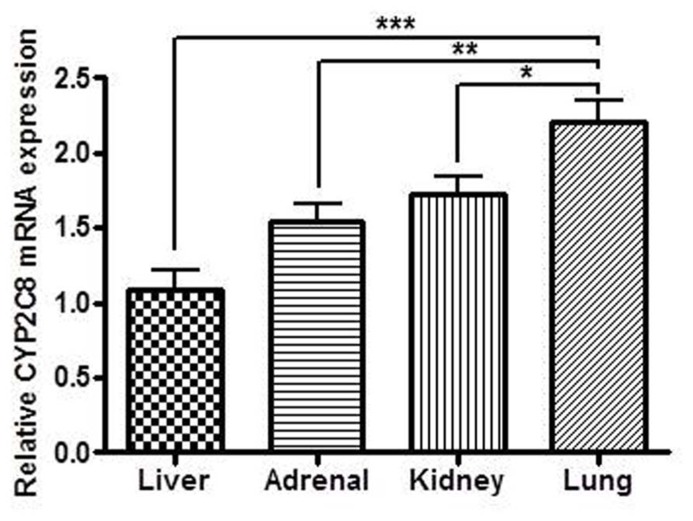
**Relative CYP2C8 mRNA expression in liver, adrenal, kidney, and lung from human fetuses (*n* = 60).** CYP2C8 mRNA expression was detected in all of the extra-hepatic tissue samples with significantly higher expression in lung compared to kidney, adrenal and liver. *p < 0.05, **p < 0.01, ***p < 0.001.

Significant differences in mRNA expression of CYP2C8 between the different organs were demonstrated with significantly higher expression in lung compared to kidney (*p* < 0.05), adrenal (*p* < 0.01), and liver (*p* < 0.001). The relative mRNA levels were in liver (mean = 1.98 ± 1.34), kidneys (mean = 3.38 ± 1.42), adrenals (mean = 3.29 ± 1.78), and lungs (mean = 4.58 ± 1.79). When performing the statistical analysis the samples were paired, thus the results in the figure represents the results from tissues from 32 fetuses (**Figure 5**).

Since the expression of CYP2C9 was found only in one kidney, two adrenal specimens, and in none of the lung samples no statistical analysis was performed.

The relationship between the mRNA expression of CYP2C8 and different organ/the age of the mother/the gestational age of the fetus/smoking was investigated. No significant correlations or differences were found (data not shown).

## DISCUSSION

Here we show that CYP2C8 and CYP2C9 gene expression in human fetus is very low, 0.70 and 0.25% of the expression found in adult livers. This result is in agreement with previous findings reporting that CYP2C9 protein in the first trimester is about 1% of the protein concentration in adults (Hines, 2007). Interestingly, hepatic CYP2C8 was approximately three times more abundant in the fetuses than CYP2C9 transcripts. This in concordance with a previous study proposing that CYP2C8 is the main CYP enzyme in the fetal liver (Hakkola et al., 1994). The situation in adult livers appears to be the opposite, where CYP2C9 is expressed at twofold higher level than CYP2C8 (Lapple et al., 2003).

In addition to CYP2C8 mRNA being more abundant than CYP2C9 mRNA in the fetal liver, CYP2C8 was found in all extra-hepatic fetal specimens examined. This was not the case for CYP2C9, which was only detectable in isolated samples of kidney and adrenals, and absent in the fetal lung. The reason for CYP2C8 to be more abundant in the early pregnancy is not known. It is possible that CYP2C8 may be required in the metabolism of endogenous compounds such as retinoic acids, known to be more specific for CYP2C8 (Marill et al., 2000), and hence protect the human fetus against retinoic acid-induced embryotoxicity.

CYP2C9 gene expression in the liver was the only transcript that correlated with gestational age. Koukouritaki et al. (2004) showed that CYP2C9 protein expression increases with advancing gestation. In the third trimester the CYP2C9 protein expression has increased from 1% in first and second trimester to reach 10% of adult levels. It is conceivable that CYP2C9 expression is induced and more important later in pregnancy. Furthermore it has been shown that CYP2C9 are further induced after birth (Hines, 2007).

The co-expression of hepatic CYP2C8 and CYP2C9 in the fetuses and adults, but not in the extra-hepatic specimens investigated, indicates that they may be transcriptionally regulated to some degree by the same liver specific factors throughout the development. HNF4, CAR, PXR, and AHX are transcription factors known to be involved in the hepatic expression of several CYPs including CYP2C8/9. A strong correlation between CYP2C9 and CYP2C8 mRNA levels in adult livers has also been observed by others (Wortham et al., 2007; Naraharisetti et al., 2010).

A large inter-individual variation in CYP2C8/9 gene expression in both the adults (70- and 220-fold, respectively) and fetal (approximately 20-fold) subjects was observed. In adults the inter-individual variation for CYP2C8 and CYP2C9 expression has been found to be in the same range as observed here, in Naraharisetti et al. (2010), Rodriguez-Antona et al. (2001), and Wortham et al. (2007), whereas this is the first time the inter-individual variation in early pregnancy has been studied. The large variability in CYP2C8/9 expression may be determined by hormonal status, diet, smoking, and drug exposure. However, there was no correlation between sex and CYP2C8/9 mRNA expression in the adult samples. Sex was not determined at the collection of fetus specimens. We could not discern any correlation with maternal smoking and CYP2C8/9 expression in this study.

The mRNA levels of CYP2C8/9 have been shown to correlate with the total activity (Rodriguez-Antona et al., 2001; Yang et al., 2010). In agreement with our gene expression results, the activity was lower in a pooled fetal sample compared to the activity in the adult sample. Ibuprofen is extensively metabolized to hydroxylated metabolites. Ibuprofen preparations contain a mixture of R-(–)- and S-(+)- ibuprofen. *S*-Ibuprofen is hydroxylated to 2-OH-ibuprofen mainly by CYP2C9, whereas CYP2C8 is the main enzyme involved in the hydroxylation of *R*-ibuprofen (Hamman et al., 1997). Here we analyzed the 2-OH-ibuprofen formation and consequently mainly the CYP2C9 activity. Minor contribution of other CYPs could be the reason why the difference in CYP2C9 activity between adult and fetus was not as large as the difference in mRNA expression. CYP3A4 has been shown to be involved in *S*-ibuprofen 2-OH-ibuprofen activity *in vitro* (Chang et al., 2008), whereas the involvement of the specific fetal CYP3A7 enzyme has not been studied. As expected, a disadvantage with this study is that the fetal specimens collected were not sufficiently large to conduct activity measurements on individual samples.

The embryonic period studied here (5–12 weeks) is the most crucial in terms of structural malformations. Drug exposure during this time may result in congenital malformations and/or growth abnormalities (Blumenfeld et al., 2010). In view of the increasing use of prescribed drugs and OTC drugs in pregnancy more knowledge in fetal drug metabolism enzyme is needed to cast light on mechanisms of drug and xenobiotic induced fetal toxicity, teratogenicity, and pharmacological response to drug treatment in pregnancy.

In conclusion, we have shown that CYP2C9 and CYP2C8 mRNA levels, as well as CYP2C9 activity are higher in adult livers as compared to fetal livers and that the tissue distribution of CYP2C8 and CYP2C9 is widely different. Our results indicate that CYP2C8 plays a more important physiological role than CYP2C9 in the first trimester, since it was more abundant in the liver and were found consistently in extra-hepatic tissues such as adrenal, kidney, and lungs.

## Conflict of Interest Statement

The authors declare that the research was conducted in the absence of any commercial or financial relationships that could be construed as a potential conflict of interest.
